# 

**Published:** 1884-09

**Authors:** 


SEPTEMBER, 1884.

### THE AMERICAN JOURNAL OF DENTAL SCIENCE.

EDITED BY

F. J. S. GORGAS, M. D., D. D. S.

Vol. 18. THIRD SERIES. No. 5.

BALTIMORE:
SNOWDEN & COWMAN.
LONDON:
TRUBNER & CO., 60 PATERNOSTER ROW.
1883.

PUBLISHED MONTHLY.

$2.50 PER ANNUM.

PAYABLE IN ADVANCE.

